# Editorial: Epigenetic Regulation in Renal Development, Physiology and Disease

**DOI:** 10.3389/fphys.2021.818190

**Published:** 2022-01-05

**Authors:** Juan Jin, Haiyong Chen, Xiao-Ming Meng

**Affiliations:** ^1^Inflammation and Immune Mediated Diseases Laboratory of Anhui, The Key Laboratory of Anti-inflammatory of Immune Medicines, Ministry of Education, School of Pharmacy, Anhui Institute of Innovative Drugs, Anhui Medical University, Hefei, China; ^2^School of Basic Medicine, Anhui Medical University, Hefei, China; ^3^Li Ka Shing Faculty of Medicine, School of Chinese Medicine, The University of Hong Kong, Pokfulam, Hong Kong SAR, China

**Keywords:** epigenetics, DNA methylation, histone modification, N6-methyladenosine (m6A) RNA methylation, long non-coding RNAs

Epigenetic mechanisms regulate heritable phenotype changes without altering DNA sequence. In this manner, fine-tuning of biological processes is usually achieved in response to environmental stimuli. Epigenetic regulations not only contribute to kidney physiological functions but also kidney diseases (Guo et al., 2019). This Research Topic aimed to summarize the current knowledge of epigenetic modifications on renal development, physiology and pathology in kidneys, as well as epigenetic regulations on cellular metabolism, inflammation and apoptosis, and intracellular signals.

Epigenetic regulations involve covalent modification of DNA or histone proteins, and RNA interference by non-coding RNAs which modulate gene/protein expression. DNA methylation is a common type of epigenetic modification that reversibly affects gene expression without changes in the sequence of nucleotides (Ginder and Williams, 2018; Grimm et al., 2019). Chen et al. recently demonstrated that DNA methylation occurring in peripheral immune cells profoundly contributes to development of kidney diseases (Mok et al., 2016; Chen et al., 2019; Klumper et al., 2020). Chen et al. reviewed that change of DNA methylation sustains for a long time in immune cells and modulates gene expression in the circulating immune cells even after the cells migrate from the circulation into the affected kidney. The aberrant DNA methylation in the immune cells was summarized in different kidney diseases, including lupus nephritis, IgA nephropathy, hypertensive nephropathy, and diabetic kidney diseases. Potential treatment of CKD targeting on DNA methylation is highlighted in the article.

Histones are highly conserved proteins with positive charge which package with negatively charged DNA into highly condensed and ordered chromatin structure units called nucleosomes (Kimura, 2013). Methylation is one of the major forms of histone modification (Kooistra and Helin, 2012). Li et al. focused on the functions of a histone methyltransferase in renal diseases. They thoroughly reviewed histone methyltransferase EZH2 that catalyzes the addition of methyl groups to histone H3 at lysine 27 and leads to gene silencing in different kidney injuries, such as acute kidney injury (AKI), renal fibrosis, diabetic nephropathy, lupus nephritis, and renal transplantation rejection. Their article summarizes the pathological roles of EZH2 in kidney diseases and highlights EZH2 as a potential therapeutic target for kidney diseases.

Epigenetic changes of functional proteins could serve as epigenetic markers to predict the progression and prognosis of a disease progression. You et al. identified a set of clinically relevant cancer-associated fibroblasts-related methylation-driven genes, NAT8, TINAG, and SLC17A1 in kidney renal clear cell carcinoma (KIRC). Methylation levels of these genes are highly correlated with the severity of KIRC. Methylation levels of the gene panel could be used as promising biomarkers to predict the progression and prognosis of KIRC.

N6-methyladenosine (m6A) is the most abundant modification which regulates post-transcriptional RNA on mRNAs. It is involved in various physiological and pathological processes, such as metabolism, inflammation, and apoptosis. Shen et al. presented that differentially m6A methylated genes are enriched in cisplatin-induced kidney injury and berberine, a chemical compound, attenuates AKI by regulating differentially methylated genes. Shi et al. indicated that variability of m6A methyltransferase METTL3 is significantly increased in clear cell renal cell carcinoma (ccRCC) which regulates translation of ABCD1, an ATP-binding cassette (ABC) transporter of fatty acids, in an m6A-dependent manner. Thus, METTLE3 promotes ccRCC progression via m6A modification-mediated translation of ABCD1. METTL3, as an m6A methyltransferase, plays an essential role in the development and progression of diseases. Nevertheless, m6A modifications by METTL3 in kidney diseases remain largely unclear. Comprehensive and systematic functions of METTL3 on post-translational modifications could be explored by conditional knockout of METTL3 from kidney in mice with kidney disease models, since METTL3 knockout mouse is embryonic lethal (Geula et al., 2015). These findings should be further verified in clinics. Specific METTL3 inhibitors may be developed for the relevant kidney diseases.

Epigenetic regulation also involves RNA interference by non-coding RNAs. Long non-coding RNAs (LncRNA) are previously reported to be regulators for multiple cellular processes and disease progresses, e.g., cell differentiation, cell proliferation, and apoptosis (Wang et al., 2017; Villa et al., 2019). Yuan et al. verified that downregulation of LncRNA H19 promotes cell proliferation, inhibits cell apoptosis, and suppresses multiple inflammatory cytokine expressions in hypoxia/reoxygenation-treated human renal proximal tubular cells by regulating the miR-130a/BCL2L11 pathway. Deng et al. also demonstrated LncRNA *MEG3* is involved in pyroptosis of renal proximal tubular cells (TECs) in lipopolysaccharide-induced AKI by regulating the miR-18a-3p/GSDMD pathway. This study showed that LncRNA might display an critical role in the pathogenesis of sepsis-related AKI through regulating pyroptosis of TECs.

In sum, epigenetic mechanisms including modification of DNA, histone proteins, or RNA interference by non-coding RNAs are designated as biochemical switches which turn on/off gene expression without affecting the DNA sequence. The manuscripts in this Research Topic provide a broad overview of the latest research investigating epigenetic regulation and relevant therapeutic potentials for diagnosis and treatments of renal diseases.

## Author Contributions

All authors listed have made a substantial, direct, and intellectual contribution to the work and approved it for publication.

## Funding

This work was supported by the National Natural Science Foundation of China (Grant Nos. 81970584 and 82100727), Promotion plan of basic and clinical cooperative research in Anhui Medical University (Grant Nos. 2019xkjT014 and 2020xkjT016), the Open Fund of Inflammation and Immune Mediated Diseases Laboratory of Anhui Province (Grant No. IMMDL202002), and Provincial Natural Science Foundation (Grant No. 1908085QH377).

## Conflict of Interest

The authors declare that the research was conducted in the absence of any commercial or financial relationships that could be construed as a potential conflict of interest.

## Publisher's Note

All claims expressed in this article are solely those of the authors and do not necessarily represent those of their affiliated organizations, or those of the publisher, the editors and the reviewers. Any product that may be evaluated in this article, or claim that may be made by its manufacturer, is not guaranteed or endorsed by the publisher.
